# Association of atherogenic index of plasma with cardiovascular disease mortality and all-cause mortality in the general US adult population: results from NHANES 2005–2018

**DOI:** 10.1186/s12933-024-02359-z

**Published:** 2024-07-16

**Authors:** Minghui Qin, Bo Chen

**Affiliations:** 1 Department of Traditional Chinese Medicine, Xiangyang Central Hospital, Affiliated Hospital of Hubei University of Art and Science, Xiangyang, Hubei China; 2 Center for Clinical Evidence-Based and Translational Medicine, Xiangyang Central Hospital, Affiliated Hospital of Hubei University of Arts and Science, Xiangyang, Hubei China; 3 Department of Endocrinology, Affiliated Hospital of Hubei, Xiangyang Central Hospital, University of Arts and Science, Xiangyang, Hubei China

## Abstract

**Background:**

The atherogenic index of plasma (AIP) is a critical metric for predicting cardiovascular outcomes. However, its associations with cardiovascular disease mortality (CVM) and all-cause mortality (ACM) remain unclear. This study aims to elucidate the relationship between baseline AIP levels and CVM and ACM among a broad cohort of US adults.

**Methods:**

Utilizing data from the National Health and Nutrition Examination Survey (2005–2018), we analyzed 18,133 adults aged ≥ 18. Baseline triglycerides and high-density lipoprotein cholesterol levels were measured to calculate the AIP. Mortality outcomes were determined through linkage with the National Death Index database, with follow-up through December 31, 2019. Multivariable Cox proportional hazard models examined the associations between baseline AIP and mortality risks. Additionally, restricted cubic splines were utilized to investigate potential non-linear relationships, with subgroup analyses conducted across strata defined by age, gender, body mass index, diabetes, hypertension, and metabolic syndrome to assess variability in these associations.

**Results:**

Over a median 95.0-month follow-up, there were 1870 all-cause deaths and 579 cardiovascular disease-related deaths. Our findings indicate a J-shaped association between the AIP and ACM (threshold = 0.0905); specifically, when baseline AIP exceeded 0.0905, a significant positive association with ACM emerged (hazard ratio, HR (95% confidence interval, CI): 1.61(1.08–2.37)). However, after adjusting for confounders, the relationship between AIP and CVM was not statistically significant (HR 1.31, 95% CI 0.93–1.86). Notably, in the 40–60-year age group, AIP was significantly positively associated with ACM and CVM, with HRs and 95% CIs of 1.51 (1.08v2.10) and 2.63 (1.39–4.98), respectively.

**Conclusions:**

A J-shaped relationship was observed between baseline AIP levels and ACM within the general US population, with a threshold of 0.0905. Moreover, AIP could potentially be an effective predictor for future ACM or CVM, particularly among individuals aged 40–60. Further investigation is warranted to corroborate these findings.

**Supplementary Information:**

The online version contains supplementary material available at 10.1186/s12933-024-02359-z.

## Background

The Atherogenic Index of Plasma (AIP) is a novel biomarker reflecting the balance of pro-atherosclerotic and anti-atherosclerotic lipoproteins in the blood [1]. As AIP considers the interaction between different lipid components that promote the development of atherosclerosis, some epidemiological studies have demonstrated that AIP can reflect atherogenic dyslipidemia more effectively than traditional lipid markers (total cholesterol, triglycerides, high-density lipoprotein cholesterol, and low-density lipoprotein cholesterol) [2, 3]. It has been proven to be an essential indicator reflecting lipid metabolism abnormalities and predicting cardiovascular outcome risks [4], making it a potential tool for assessing the risk of cardiovascular disease [5]. A recent series of studies has also shown that an increase in the AIP is associated with a higher risk of cardiovascular disease [6], coronary heart disease [7], peripheral arterial disease [1], and stroke [2].

Given that AIP can more comprehensively capture the potential of lipoproteins to cause atherosclerosis, AIP is expected to become further a valuable tool for assessing adult cardiovascular disease mortality (CVM) and all-cause mortality (ACM). However, the relationship between the AIP and CVM or ACM remains controversial. To date, only a few observational studies have focused on exploring the potential utility of AIP in predicting CVM and ACM in different populations [4, 8–10]. Some studies have reported no significant relationship between the AIP and CVM and ACM in the East Asian populations [4], while others documented a U-shaped relationship in hypertension patients [8] or a positive association in patients with diabetes [9]. Accordingly, the controversy surrounding the AIP has hindered its clinical applicability. Meanwhile, the relationship between the AIP and CVM or ACM in the general white population is still unclear. Given that the AIP is an effective and inexpensive parameter, further exploration of the associations between the AIP and CVM and ACM is crucial to promote its clinical use and enhance overall survival.

This study aims to utilize data from the National Health and Nutrition Examination Survey (NHANES) 2005–2018 to explore the relationship between the AIP and CVM as well as ACM in American adults, thereby filling the knowledge gap about the AIP index in predicting CVM as well as ACM in a nationally representative sample.

## Methods

### Study participants and design

This study utilized data from a large cross-sectional survey conducted by the Centers for Disease Control and Prevention in the United States, namely NHANES. NHANES aims to assess the health status of the U.S. population using a nationally representative sample. The Institutional Review Board of the National Center of Health Statistics approved the initial survey protocol. All participants provided an informed consent form and complied with the Declaration of Helsinki [11]. Seven waves of NHANES (2005–2018) provided data for 42,144 participants aged 18 and older. 23,969 participants were excluded because they did not take measurements for triglycerides (TG) and high-density lipoprotein cholesterol (HDL-C), and 317 participants with abnormal AIP values were excluded. Additionally, 42 individuals were excluded due to the lack of all-cause mortality dates and medical condition data (Figure S1). Ultimately, this study was based on 18,133 individuals aged 18 and older who completed a series of questionnaire surveys, on-site assessments, and laboratory examinations at the Mobile Examination Center or their homes.

### Baseline indicators measurement assessment

In the study design, trained investigators gathered demographic information (e.g., age, gender, and marital status), lifestyle factors (e.g., alcohol consumption and smoking status), family history of diabetes, and history of hypertension from the participants using a standardized NHANES demographic and health questionnaire. All participants’ height, waist circumference (WC), weight, and blood pressure were measured by examination personnel at the Mobile Examination Center. Blood pressure was measured three times to obtain an average value.

Race was categorized as non-Hispanic Black, non-Hispanic white, Mexican American, other Hispanic, and other Race. Body mass index (BMI) was computed using a standard formula. Marital status was categorized as married or living with a partner, widowed or divorced, and never married. Education status was categorized as college or higher education, associate degree, high school graduate, and 11th grade or below. Smoking status was categorized as never smoked (defined as smoking fewer than 100 cigarettes in a lifetime), former smoker (smoked more than 100 cigarettes but current not smoking), and current smoker (smoked more than 100 cigarettes and currently smoking occasionally or daily) [12]. Drinking status was categorized as heavy drinking (defined as consuming ≥ 3 drinks per day for women and ≥ 4 drinks per day for men in the past 12 months), moderate drinking (defined as consuming 2–3 drinks per day for women and 3–4 drinks per day for men in the past 12 months), light drinking (in the past 12 months, women consumed an average of ≤ 2 drinks per day, and men consumed an average of ≤ 3 drinks per day), and never drinking (did not meet the above criteria) [13]. Diabetes was defined as meeting one of the following criteria: (1) self-reported diagnosis of diabetes by a doctor, (2) taking hypoglycemic drugs, (3) glycated haemoglobin (HbA1c) ≥ 6.5%, or (4) fasting blood glucose ≥ 7.0 mmol/L. Similarly, hypertension was defined as the self-reported diagnosis of hypertension by a doctor, taking antihypertensive medications, or an average systolic blood pressure ≥ 140 mmHg or average diastolic blood pressure ≥ 90 mmHg. Incident metabolic syndrome (MetS) was defined as meeting ≥ 3 criteria: (1) abdominal obesity (waist circumference > 80 cm for women, > 90 cm for men), (2) elevated fasting blood glucose (≥ 5.6mmol/L, physician’s diagnosis of diabetes, or diabetic medication use), (3) hypertriglyceridemia (plasma triglycerides > 150 mg/dL), (4) low HDL-C (plasma HDL-C < 50 mg/dL for women, < 40 mg/dL for men), and (5) elevated blood pressure (SBP > 130 mmHg, DBP > 85 mmHg, physician’s diagnosis of hypertension, or antihypertensive medication use) [14].

Fasting venous blood samples were collected according to NHANES quality assurance and quality control protocols. A Beckman 5,800 automatic biochemical analyzer was used to examine common biochemical markers, including TG, total cholesterol (TC), low-density lipoprotein cholesterol (LDL-C), HDL-C, alanine aminotransferase (ALT), urea nitrogen (BUN), serum creatinine (Scr), HbA1c level, and aspartate aminotransferase (AST). The AIP was computed using the formula: log10 (TG/HDL-C) [1]. Based on the AIP value, all individuals were categorized into four quartiles: quartile 1 (−1.25, − 0.29), quartile 2 (− 0.29, − 0.07), quartile 3 (− 0.07, 0.15), quartile 4 (0.15, 1.45).

### Mortality outcomes of the study population

In this survey, the endpoints were CVM and ACM. Mortality data were obtained by linking the National Death Index (NDI) database of the National Center for Health Statistics (NCHS) and the public-use death records as of December 31, 2019, using a probability-matching algorithm [15]. ACM was defined based on the International Classification of Diseases, 10th revision (ICD-10). Among the nine detailed categories of underlying and contributing causes of death provided in the data, heart disease and cerebrovascular disease were used to assess CVM (ICD codes I00-I09, I11, I13, I20-I51, I60-I69) [16]. Follow-up time was calculated from the baseline interview date to the date of death or the study end date (December 31, 2019, whichever occurred first).

### Statistical analysis

We performed multiple imputations using chained equations to mitigate potential bias from missing data,. For continuous variables, descriptive statistics are presented as median (interquartile range) or mean ± standard deviation; categorical variables are presented as frequency (percentage). Differences between groups were analyzed using the chi-square test, Kruskal-Walli’s test, or analysis of variance, as appropriate. The Kaplan-Meier method was employed to compare survival and cumulative event rates. Kaplan-Meier hazard ratios (HRs) for mortality were analyzed using the log-rank test.

Using tolerance and variance inflation factors, we assessed collinearity between the AIP and other covariates [17]. During the follow-up period, ACM and CVM were calculated for each AIP quartile group. Multivariable Cox regression models were employed to evaluate the impact of AIP on CVM and ACM. We plotted Schoenfeld residuals over time before modelling to validate the proportional hazards assumption. Based on the STROBE statement [18], two stepwise adjusted models were applied to assess the associations between AIP (and its quartiles) and mortality: Model I was unadjusted; Model II adjusted for gender, age, race, education, family income-poverty ratio, BMI, smoking status, drinking status, and LDL_C. We used the median of each AIP quartile to test for linear trends with the mortality. Additionally, the AIP was analyzed as a continuous variable to assess the risk of death per one standard deviation (1 SD) increase. Restricted cubic splines (RCS) with 4-knot were employed in the Cox model to evaluate potential non-linear dose-response relationships between AIP and mortality. If non-linearity was detected, we estimated the threshold by maximizing the likelihood across all possible values. A two-segment Cox hazards model was then used to analyze mortality risk associations above and below the threshold.

Subgroup analyses were conducted to assess whether the association between AIP and CVM or ACM were modified by age (< 40, 40–60, and ≥ 60 years), sex, BMI (< 25, 25–29, and > 29 kg/m2), diabetes, hypertension, and metabolic syndrome). Two sensitivity analyses enhanced result reliability: (1) To mitigate reverse causality, we repeated the analysis after excluding baseline cancer and severe cardiovascular disease patients; (2) We calculated the E-value to quantify the minimum strength of association that an unmeasured confounder would require with both AIP and mortality to explain away the observed associations [19]. Analyses were performed using Empower^®^2.0, R-4.3.0, and SAS 9.4 (SAS Institute, Cary, NC), with *P* < 0.05 considered statistically significant.

## Results

### Baseline characteristics by AIP quartiles

The analytic sample included 18,133 participants (mean age 48.15 ± 18.75 years; 48.62% male). Over a median 95.0-month follow-up (interquartile range: 54.2—135.0 months), 1,870 individuals (10.32%) died, including 579 (30.96%) from cardiovascular causes and 1,291 (69.03%) from non-cardiovascular causes. AIP was a normal distribution in this cohort (Figure S2). Across increasing AIP quartiles (Table 1), significant differences were observed for age, BMI, gender, race, marital status, education level, smoking, and drinking status (all *p* < 0.05). Higher AIP was associated with being males, non-Hispanic whites, married/cohabiting, having ≤ high school education, smoking, heavy drinking, diabetes, and hypertension (all *p* < 0.05). Biochemical indicators also differed significantly across AIP quartiles (Table 2), with the highest quartile exhibiting elevated glycohemoglobin, ALT, AST, SBP, DBP, LDL-C, TC, TG, Scr, GGT, uric acid, and FPG compared to the lowest quartile (all *P* < 0.05).

**Table 1:** Baseline characteristics stratified by AIP quartiles

	AIP quartiles					*P*-value
	Overall	Q1 (− 1.25, − 0.29)	Q2 (− 0.29, − 0.07)	Q3 (− 0.07, 0.15)	Q4 (0.15, 1.45)	
No. of subjects	18,133	4,515	4,556	4,542	4,520	
Age, years	48.00 (32.00–63.00)	42.00 (28.00–61.00)	48.00 (31.00–64.00)	50.00 (34.00–65.00)	50.00 (36.00–63.00)	< 0.001
Family income-poverty ratio	2.04 (1.08–3.97)	2.20 (1.13–4.21)	2.10 (1.09–4.08)	2.04 (1.09–3.84)	1.83 (1.03–3.61)	< 0.001
BMI	28.90 (6.99)	26.15 (6.41)	28.30 (6.75)	30.06 (7.07)	31.29 (6.60)	< 0.001
Gender						< 0.001
Male	8,816 (48.62%)	1,646 (36.46%)	2,063 (45.28%)	2,309 (50.84%)	2,798 (61.90%)	
Female	9,317 (51.38%)	2,869 (63.54%)	2,493 (54.72%)	2,233 (49.16%)	1,722 (38.10%)	
Race						< 0.001
Non-Hispanic Black	3,773 (20.81%)	1,467 (32.49%)	1,049 (23.02%)	784 (17.26%)	473 (10.46%)	
Non-Hispanic White	7,582 (41.81%)	1,656 (36.68%)	1,881 (41.29%)	1,922 (42.32%)	2,123 (46.97%)	
Mexican American	2,968 (16.37%)	478 (10.59%)	719 (15.78%)	826 (18.19%)	945 (20.91%)	
Other Hispanic	1,789 (9.87%)	346 (7.66%)	426 (9.35%)	495 (10.90%)	522 (11.55%)	
Other Race	2,021 (11.15%)	568 (12.58%)	481 (10.56%)	515 (11.34%)	457 (10.11%)	
Marital Status						< 0.001
Married or living with partner	10,547 (58.16%)	2,310 (54.09%)	2,570 (58.15%)	2,772 (61.69%)	2,895 (64.56%)	
Widowed or divorced	1,372 (7.57%)	316 (7.51%)	352 (8.13%)	387 (8.73%)	317 (7.15%)	
Never married	6,214 (34.27%)	1,889 (38.40%)	1,634 (33.72%)	1,383 (29.59%)	1,308 (28.29%)	
Education						< 0.001
College or above	4,617 (25.46%)	7,870 (19.14%)	1,069 (23.58%)	11,243 (27.02%)	1,435 (31.52%)	
Associate (AA) degree	4,071 (22.45%)	905 (20.38%)	1,045 (23.20%)	1,063 (23.74%)	1,058 (23.61%)	
High school graduates	5,334 (29.42%)	1,434 (31.02%)	1,358 (29.06%)	1,279 (27.98%)	1,263 (27.83%)	
Below grade 11	4,111 (22.67%)	1,306 (29.46%)	1,084 (24.16%)	9,957 (21.26%)	764 (17.04%)	
Smoking status						< 0.001
Never	10,172 (56.10%)	2,884 (63.66%)	2,691 (58.82%)	2,464 (53.90%)	2,133 (46.83%)	
Former	4,347 (23.97%)	933 (20.71%)	1,019 (22.66%)	1,142 (25.29%)	1,253 (27.92%)	
Now	3,614 (19.93%)	698 (15.63%)	846 (18.52%)	936 (20.81%)	1,134 (25.25%)	
Alcohol						< 0.001
Heavy	3,098 (17.08%)	690 (15.28%)	746 (16.37%)	796 (17.53%)	866 (19.16%)	
Moderate	2,397 (13.22%)	729 (16.15%)	619 (13.59%)	570 (12.55%)	479 (10.60%)	
Mild	5,289 (29.17%)	1,360 (30.12%)	1,336 (29.32%)	1,291 (28.42%)	1,302 (28.81%)	
Never	7,349 (40.53%)	1,736 (38.45%)	1,855 (40.72%)	1,885 (41.50%)	1,873 (41.44%)	
Diabetes						< 0.001
Yes	3,523 (19.43%)	458 (10.14%)	692 (15.49%)	1,018 (22.41%)	1,355 (29.98%)	
No	14,610 (80.57%)	4,057 (89.86%)	3,864 (84.81%)	3,524 (77.59%)	3,165 (70.02%)	
Hypertension						< 0.001
Yes	7,353 (40.55%)	1,400 (31.01%)	1,721 (37.77%)	2,026 (44.61%)	2,206 (48.81%)	
No	10,780 (59.45%)	3,115 (68.99%)	2,835 (62.23%)	2,516 (55.39%)	2,314 (51.19%)	
All-cause death						< 0.001
Yes	1,870 (10.31%)	338 (7.49%)	475 (10.43%)	515 (11.34%)	542 (11.99%)	
No	16,263 (89.69%)	4,177 (92.51%)	4,081 (89.57%)	4,027 (88.66%)	3,978 (88.01%)	
CVD death						< 0.001
Yes	579 (3.19%)	94 (2.08%)	142 (3.12%)	168 (3.70%)	175 (3.87%)	
No	17,554 (96.81%)	4,421 (97.92%)	4,414 (96.88%)	4,374 (96.30%)	4,345 (96.13%)	

Values were expressed as mean (standard deviation) or medians (quartile interval) or n (%)

AIP: atherogenic index of plasma; BMI: body mass index; CVD: cardiovascular disease

**Table 2:** Baseline laboratory characteristics stratified by AIP quartiles

	AIP quartile				*P*-value
	Q1 (− 1.25, − 0.29)	Q2 (− 0.29, − 0.07)	Q3 (− 0.07, 0.15)	Q4 (0.15, 1.45)	
Glycohemoglobin, %	5.47 (0.70)	5.62 (0.92)	5.82 (1.15)	6.05 (1.39)	< 0.001
ALT, U/L	17.00 (14.00–23.00)	19.00 (15.00–25.00)	21.00 (16.00–29.00)	25.00 (18.00–35.00)	< 0.001
Albumin, g/L	42.28 (3.44)	41.94 (3.63)	41.66 (3.67)	41.88 (3.74)	< 0.001
AST, U/L	21.00 (18.00–26.00)	22.00 (19.00–26.00)	22.00 (19.00–27.00)	24.00 (20.00–29.00)	< 0.001
TBil, umol/L	11.97 (8.55–13.68)	11.97 (8.55–15.39)	10.26 (8.55–13.68)	11.97 (8.55–13.68)	0.037
SBP, mmHg	119.82 (18.43)	122.64 (19.11)	124.50 (18.59)	125.71 (17.88)	< 0.001
DBP, mmHg	67.21 (12.54)	68.08 (13.05)	69.13 (12.91)	70.93 (13.24)	< 0.001
BUN, mmol/L	4.28 (3.57–5.36)	4.28 (3.57–5.71)	4.64 (3.57–5.71)	4.64 (3.57–5.71)	< 0.001
HDL-C, mmol/L	1.73 (1.50–2.02)	1.45 (1.27–1.66)	1.24 (1.09–1.42)	1.03 (0.91–1.16)	< 0.001
LDL-C, mmol/L	2.53 (2.04–3.10)	2.85 (2.30–3.41)	2.97 (2.38–3.62)	3.05 (2.38–3.67)	< 0.001
TC, mmol/L	4.63 (4.01–5.35)	4.76 (4.11–5.46)	4.89 (4.21–5.61)	5.20 (4.47–6.00)	< 0.001
TG, mmol/L	0.64 (0.52–0.78)	0.96 (0.82–1.14)	1.37 (1.17–1.59)	2.27 (1.86–2.97)	< 0.001
Scr, mg/dL	0.80 (0.69–0.94)	0.83 (0.70–1.00)	0.85 (0.70–1.00)	0.88 (0.73–1.02)	< 0.001
GGT (IU/L)	16.00 (12.00–23.00)	18.00 (13.00–26.00)	20.00 (15.00–31.00)	26.00 (18.00–41.00)	< 0.001
eGFR, mL/min/1.73m^2^	97.96 (81.82–113.78)	94.42 (77.89-110.43)	93.59 (76.05–109.07)	94.30 (76.43–108.88)	< 0.001
Serum iron, umol/L	14.70 (10.90–19.20)	15.00 (11.50–19.30)	15.00 (11.30–19.20)	15.00 (11.50–19.30)	0.06
Lactate dehydrogenase, U/L	129.00 (113.00–149.00)	129.00 (114.00-149.00)	129.00 (112.00–148.00)	128.00 (113.00–147.00)	0.546
Serum insulin, pmol/L	39.69 (26.76–59.64)	52.98 (34.86–80.82)	67.17 (43.14–104.45)	89.34 (57.54–139.27)	< 0.001
Uric acid, umol/L	279.60 (237.90–339.00)	309.30 (255.80–362.80)	333.10 (273.60–386.60)	356.90 (297.40–410.40)	< 0.001
FPG, mmol/L	5.52 (1.22)	5.79 (1.53)	6.15 (1.97)	6.72 (2.73)	< 0.001

Values were expressed as mean (standard deviation) or medians (quartile interval) or n (%)

AIP: atherogenic index of plasma; SBP: systolic blood pressure; DBP: diastolic blood pressure; TG: triglyceride; TC: total cholesterol; HDL-C: high-density lipoprotein cholesterol; LDL-C: low-density lipoprotein cholesterol; ALT: alanine aminotransferase; AST: aspartate aminotransferase; BUN: blood urea nitrogen; Scr: creatinine; TBil: Serum total bilirubin; FPG: Fasting plasma glucose; GGT: Gamma Glutamyl Transferase; eGFR: Estimated Glomerular Filtration Rate

### Associations of AIP with all-cause mortality

**Fig. 1:**
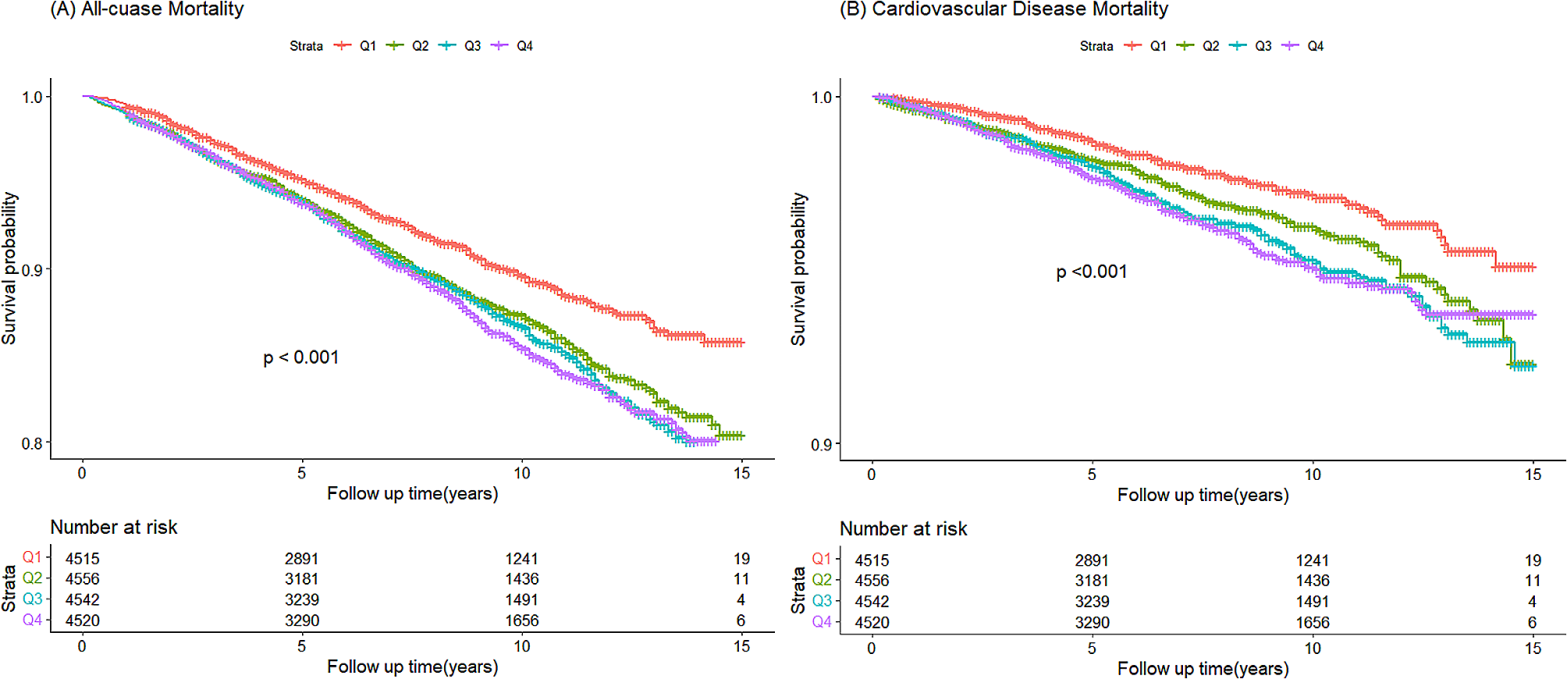
Kaplan–Meier curves depicting survival rate and the number (%) of a general population of US adults stratified by AIP quartiles. **A** All-cause mortality. **B** Cardiovascular mortality

With ACM and CVM as the dependent variables, we assessed the proportional hazards assumption by plotting the Schoenfeld residuals for the AIP over time (Figure S3) and checked for collinearity between AIP and other covariates. Results supported employing the Cox model. Collinearity diagnostics revealed variance inflation factors (VIFs) exceeding 5 for TC, TG, and HDL-C, prompting their exclusion from multivariable models (Table S1). As shown in Table 3, the unadjusted Cox analysis demonstrated significantly higher ACM across increasing AIP quartiles (hazard ratios [HRs] and 95% confidence intervals [CIs]from lowest to highest: 1.00 (reference), 1.30 (1.13, 1.49), 1.38 (1.20, 1.58), and 1.41 (1.23, 1.61), with significant differences in Kaplan-Meier curves (Fig. 1A). However, after adjusting for gender, age, race, education, family income-poverty ratio, BMI, smoking status, drinking status, and LDL-C, the highest AIP quartile was not significantly associated with ACM compared to the lowest quartile (HR: 1.07, 95% CI: 0.88–1.38, p-trend = 0.499).

**Table 3:** Hazard ratios (HR) and 95% confidence intervals (95%CI) for mortality according to the AIP quartiles

	No. of case	Person-years	Incidence density (1000 person-year)	HR (95%CI)*	
				Model I	Model II
**All-cause mortality**					
AIP				**1.40 (1.22**,** 1.60)**	1.07 (0.88, 1.38)
AIP (quartile)					
Q1	338	32277.75	10.47	Ref	Ref
Q2	475	34640.83	13.71	**1.30 (1.13**,** 1.49)**	1.06 (0.91, 1.24)
Q3	515	35239.92	14.61	**1.38 (1.20**,** 1.58)**	0.97 (0.83, 1.14)
Q4	542	36231.83	14.96	**1.41 (1.23**,** 1.61)**	1.04 (0.88, 1.22)
P-trend				**< 0.0001**	0.4991
Per 1SD increase				**1.12 (1.06**,** 1.17)**	1.02 (0.93, 1.08)
**CVD mortality**					
AIP				1.60 (1.26, 2.04)	1.31 (0.93, 1.86)
AIP (quartile)					
Q1	94	32277.75	2.91	Ref	Ref
Q2	142	34640.83	4.10	**1.40 (1.08**,** 1.82)**	1.10 (0.83, 1.47)
Q3	168	35239.92	4.77	**1.62 (1.26**,** 2.09)**	1.11 (0.84, 1.47)
Q4	175	36231.83	4.83	**1.64 (1.27**,** 2.10)**	1.21 (0.90, 1.62)
P-trend				**< 0.0001**	0.1262
Per 1SD increase				**1.16 (1.07**,** 1.26)**	1.08 (0.97, 1.82)

*The symbol bold reflected p<0.05

Model I: Non-adjusted

Model II: adjusted for gender, age, race, education, family income-poverty ratio, BMI, smoking status, drinking status, low-density lipoprotein cholesterol

Abbreviations: HR: hazard ratios; CI: confidence interval; other abbreviations as in Table 1

**Fig. 2:**
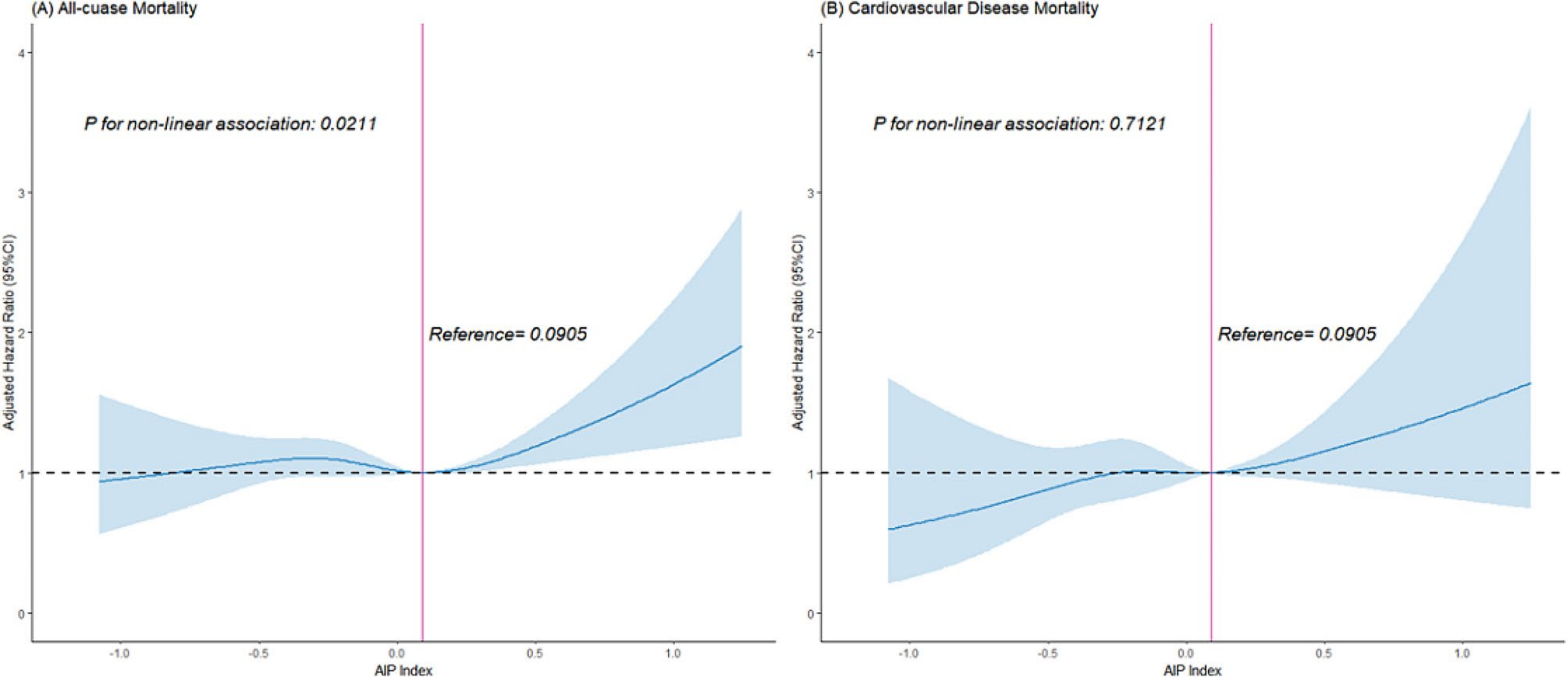
Dose-response curve of AIP and all-cause mortality (**A**) and CVD mortality (**B**) using restricted cubic splines with 4-knots. Each hazard ratio was computed with an AIP of 0.0905 as the reference. Adjusted for age, gender, race, education, family income-poverty ratio, BMI, smoking status, drinking status, and LDL_C. The solid line and blue area represent the estimated values and their corresponding 95% CIs, respectively. AIP: atherogenic index of plasma

 Further analyses using restricted cubic spline revealed a non-linear association between AIP and ACM (*p* = 0.021): when baseline AIP < 0.0905, AIP was not significantly associated with ACM risk (HR: 0.86, 95% CI: 0.61–1.19); however, when baseline AIP ≥ 0.0905, AIP exhibited a positive association with ACM risk (HR: 1.61, 95% CI: 1.08–2.37) (Fig. 2A, Table 4).

**Table 4:** Result of the two-piecewise Cox regression model

	HR (95%CI)*	*P*-value
**All-cause mortality**		
Total	1.07 (0.88, 1.38)	0.2397
Fitting by two-piecewise Cox proportional risk model		
The inflection point of AIP	0.0905	
< 0.0905	0.86 (0.61, 1.19)	0.5815
> 0.0905	**1.61 (1.08**,** 2.37)**	**0.0192**
**CVD mortality**		
Total	1.31 (0.93, 1.86)	0.0572
Fitting by two-piecewise Cox proportional risk model		
The reference point of AIP	0.0905	
< 0.0905	1.19 (0.63, 2.23)	0.5835
> 0.0905	1.51 (0.73, 3.10)	0.2664

*The symbol bold reflected p<0.05

Abbreviations: HR: hazard ratios; CI: confidence

Adjusted for age, gender, race, education, family income-poverty ratio, BMI, smoking status, drinking status, and low-density lipoprotein cholesterol

### Associations of AIP with cardiovascular disease mortality

Among the 18,133 participants (138,390 person-years), 579 individuals (3.19%) died from cardiovascular disease. In the unadjusted model, higher AIP was associated with increased CVM risk (HR: 1.60, 95% CI: 1.26–2.04, *p* < 0.001) (Table 3). Kaplan-Meier curves demonstrated poorer cardiovascular survival with higher AIP quartiles (*p* < 0.001) (Fig. 1B). After multivariable adjustment, HRs and 95% CIs from lowest to highest AIP quartile were 1.00 (reference), 1.10 (0.83–1.47), 1.11 (0.84–1.47), and 1.21 (0.90–1.62), respectively (p-trend = 0.1262).

The adjusted restricted cubic spline analysis did not suggest a nonlinear relationship between AIP and CVM (*p* = 0.712, Fig. 2B). When stratified by AIP = 0.0905, AIP was not significantly associated with cardiovascular disease death risk (*p* > 0.05) (Table 4).

### Subgroup analysis

**Fig. 3:**
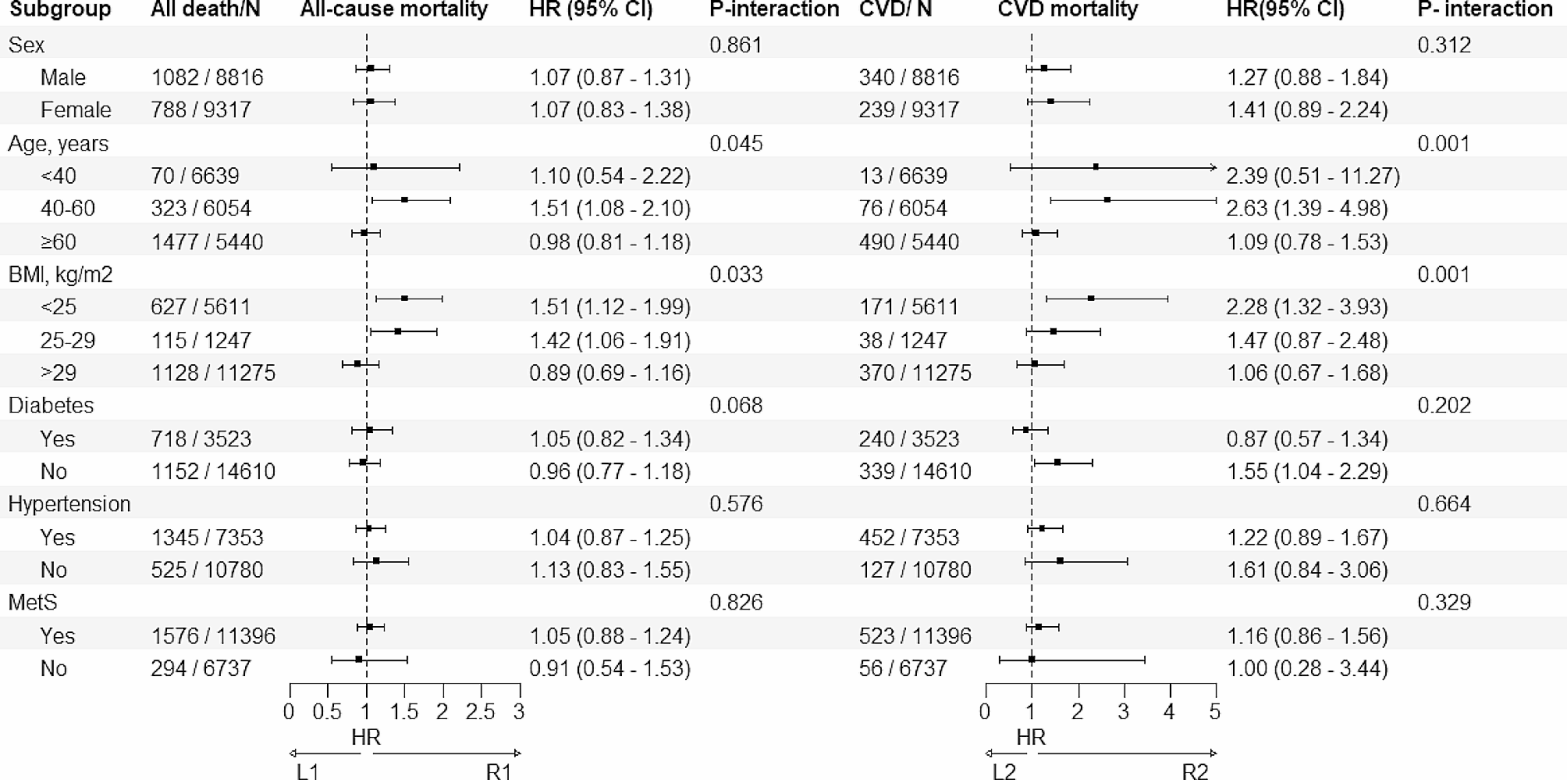
Exploratory stratified analysis of the associations between AIP and All-cause or CVD mortality

 AIP was analyzed as a continuous variable across subgroups defined by age, sex, BMI, diabetes, hypertension, and MetS. Significant positive associations were observed between AIP and CVM or ACM among patients aged 40–60 years or with BMI < 25 kg/m2, with HRs (95% CI) of 1.51 (1.08–2.10) and 1.51 (1.12–1.99) for ACM, and 2.63 (1.39–4.98) and 2.28 (1.32–3.93) for CVM, respectively. Among non-diabetic individuals, AIP exhibited a significant positive association with CVM (HR: 1.55, 95% CI: 1.04–2.29) (Fig. 3). Further subgroup analyses using an AIP threshold of 0.0905 revealed significant associations between higher AIP and ACM among those aged 40–60 years, with BMI < 25 kg/m2, diabetes, or hypertension when AIP > 0.0905 (HRs (95% CIs): 1.97 (1.06–3.67), 3.95 (1.65–9.47), 1.68 (1.05–2.69), and 1.52 (1.00–2.33), respectively) (Table S2).

**Fig. 4:**
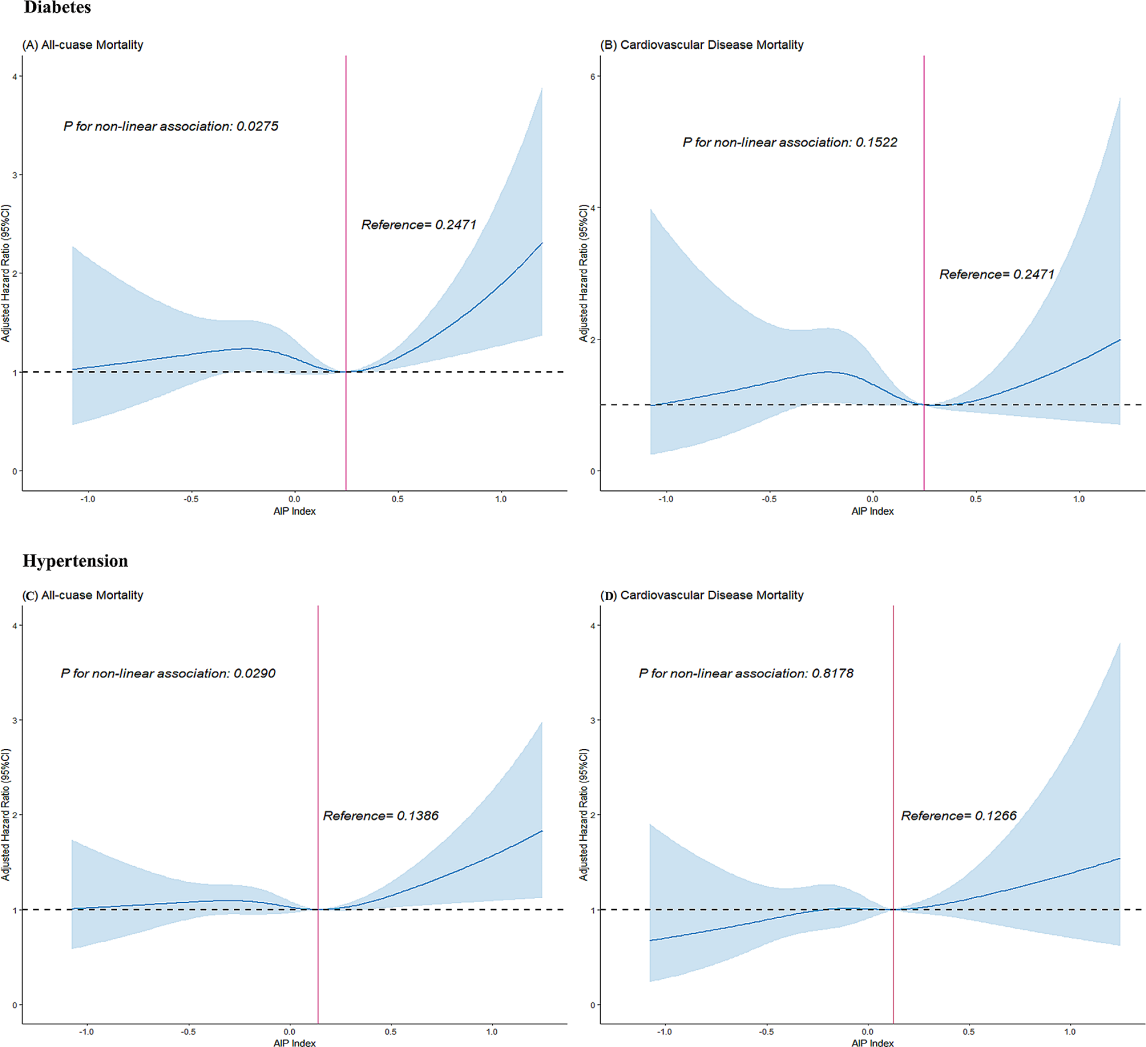
Association between AIP and all-cause mortality (**A**) and CVD mortality (**B**) in diabetes patients, with an AIP of 0.2471 as the reference. Association between AIP and all-cause mortality (**C**) and CVD mortality (**D**) in hypertension patients, with an AIP of 0.1386 as the reference. Adjusted for age, gender, race, education, family income-poverty ratio, BMI, smoking status, drinking status, and LDL_C. The solid line and blue area represent the estimated values and their corresponding 95% CIs, respectively. AIP: atherogenic index plasma

 Moreover, among individuals with diabetes or hypertension, restricted cubic spline analyses suggested non-linear relationships between AIP and ACM (*p* = 0.0275 and *p* = 0.0290), with AIP thresholds of 0.2471 and 0.1386, respectively. However, no non-linear associations between AIP and CVM were observed in these subgroups (*p* = 0.1522 and *p* = 0.8178, respectively) (Fig. 4).

### Sensitivity analysis

To assess the robustness of the AIP-mortality associations, we conducted a sensitivity analysis excluding baseline cancer and severe cardiovascular disease patients (Table S3). Results remained consistent with the primary study. Additionally, based on model 2, we computed the E-value to quantify the minimum strength of association an unmeasured confounder would require with both AIP and mortality to explain away the observed associations. The e-value for the AIP-ACM association was 1.08, while for AIP-CVM it was 1.44. These E-values suggest that relatively small unmeasured confounding effects could account for the observed hazard ratios.

## Discussion

In this relatively large, prospective, population-based national adult cohort study, we investigated the relationship between AIP and survival outcomes in the general population. Our key findings were: Firstly, after adjusting for covariates, we observed a J-shaped association (cutoff = 0.0905) between increasing AIP and ACM. Similar associations were also noted among individuals with diabetes, those aged 40–60 years, and those with a BMI ≤ 29 kg/m2. Secondly, we found that an elevated baseline AIP significantly increased the risk of CVM. However, after adjustment for traditional risk factors, the association between AIP and CVM diminished. In patients aged 40–60 years, with BMI < 25 kg/m2, or without diabetes, AIP exhibited a significant positive association with CVM.

As a novel, simple blood lipid parameter for assessing atherosclerosis risk, AIP has demonstrated utility in evaluating and predicting vascular-related diseases [20–22]. Our study involving the general White population found that AIP exhibited a J-shaped relationship with ACM. Simultaneously, after adjusting for traditional covariates, the association between AIP and CVM became insignificant, aligning with Kim et al. [4], suggesting that AIP is not associated with CVM. Notably, our study’s relatively short median 95.0-month follow-up duration might contribute to this finding, as a longer follow-up period may yield different results. However, our findings are inconsistent with three other studies. We speculate that differences in study populations, population race heterogeneity, and variations in the standardized AIP definitions may account for these discrepancies. Previous studies have demonstrated distinct lipid profiles across different population races and BMI levels [23, 24]. Tamosiunas et al. investigated the middle-aged and elderly population in Lithuanian cities and found that higher AIP level was significantly associated with increased risks of CVM in males and ACM in females [10]. In our study, which focused on the American general population over 18 years, consisting mainly of non-Hispanic Black and White individuals, racial disparities and BMI differences may partly explain the heterogeneity in results. Furthermore, our study employed the standardized AIP definition proposed by Professors Frohlich J, which may differ from the definition used in studies by Duiyimuhan et al. [8], leading to heterogeneity in results.

Our study reveals a nonlinear J-shaped association between the AIP and ACM (cutoff = 0.0905). Significantly positive associationS were observed among individuals aged 40–60 years, those with BMI ≤ 29 kg/m2, and those with diabetes or hypertension when AIP > 0.0905. These findings underscore the importance of clinicians paying particularl attention to these subgroups and individuals with AIP > 0.0905. Furthermore, our study demonstrates a significant positive association between AIP and CVM among individuals aged 40–60, aligning with previous research on cardiovascular disease and coronary syndrome patients. Min et al. demonstrated that individuals with higher AIP had an increased incidence of major cardiovascular diseases among patients aged 53–61, although no discernible difference was observed in elderly patients [25]. Similarly, chronic coronary syndrome patients under 60 years exhibited elevated AIP and increased risks of carotid atherosclerosis and carotid intima-media thickening [21]. In contrast, Kim et al. found no significant difference in the AIP regarding CVM across different age groups [4], while Fu et al. reported a significant positive association between AIP and CVM or ACM in all age groups of diabetic patients [9]. The discrepancies in these findings may be attributed to variations in the research populations. Our study investigate the relationship between AIP and mortality in different age subgroups of the general population, while Fu and colleagues primarily concentrate on the diabetic population. Notably, our study observed a positive association between AIP and CVM in patients with a BMI < 25 kg/m2 and without diabetes, contrasting with previous studies on major adverse cardiovascular events. Wang et al. found a positive association between AIP and major adverse cerebrovascular events in patients with a BMI ≤ 28 kg/m² and HbA1c > 6.5% [26], whereas Kim et al. reported a significant relationship between AIP and the risk of ischemic heart disease in non-diabetic patients [27]. Sadeghi et al. further highlighted AIP as an independent predictor of cardiovascular events in non-diabetic individuals aged > 35 years [28]. Taken together with these previous findings, our results suggest that AIP may be an effective alternative predictor of future CVM in the general population aged 40–60.

The association between AIP and mortality may stem from underlying mechanisms involving atherosclerosis and insulin resistance. Firstly, elevated plasma triglyceride levels can contribute to forming small, dense, oxidized LDL-C particles, thereby increasing the risk of atherosclerosis [29, 30]. Atherosclerosis, characterized by plaque accumulation in arterial walls, raises the likelihood of unplanned revascularization events and is closely associated with stroke and acute coronary syndrome, which are primary contributors to CVD. Previous research has suggested that the esterification rate of HDL-C and the size of lipoprotein particles may mediate the relationship between AIP and CVM [31]. Secondly, evidence indicates that insulin resistance may also play a mediating role in the risk of CVM associated with AIP. Insulin resistance diminishes the utilization of nitric oxide, thereby impairing vascular endothelial function and accelerating the progression of cardiovascular disease, ultimately leading to adverse outcomes [32]. However, it is essential to acknowledge that the precise mechanisms underlying the impact of AIP on CVM and ACM require further exploration and elucidation through additional research endeavours.

This study possesses several strengths. Firstly, it utilizes a prospective design to affirm a J-shaped association between high AIP and ACM within a sizable sample of multi-ethnic adults in the United States. Furthermore, it identifies the inflection point at 0.0905, elucidating a critical threshold for risk assessment. Moreover, the study highlights the age dependency of AIP’s predictive value for CVM, contributing valuable insights into risk stratification based on age. Secondly, the study thoroughly examines gender, age, BMI, diabetes, hypertension, and MetS-specific differences in the AIP, thereby enriching the understanding of association heterogeneity. This nuanced analysis provides valuable insights into how various demographic and clinical factors influence the relationship between AIP and mortality outcomes. However, it is crucial to acknowledge certain limitations of the study. Firstly, due to constraints imposed by the database, the relatively small number of cardiovascular disease deaths and the short follow-up duration may restrict a comprehensive evaluation of interactions. Future studies with longer-term follow-up periods are warranted to address this limitation adequately. Secondly, as an observational study, the research did not fully adjust for potential confounding factors such as eGFR and other blood indicators. However, given that AIP was not found to be associated with CVM after adjusting for traditional confounders, further adjustment may be deemed unnecessary. Nonetheless, the calculation of the E-Value reaffirmed the robustness of the study results. Thirdly, the study solely evaluates the association between baseline AIP and mortality. Future research examining changes in the AIP during follow-up and its relationship with mortality outcomes could provide additional insights and enhance the significance of the study findings.

## Conclusion

Our research results indicate that a J-shaped relationship was observed within the general US population between baseline AIP levels and ACM (cutoff = 0.0905). When baseline AIP ≥ 0.0905, AIP demonstrated a significant positive association with ACM risk. Moreover, the simple and inexpensive AIP index could potentially serve as an effective predictor for future ACM or CVM, particularly among individuals aged 40–60. Further investigation is warranted to corroborate these findings.

### Supplementary Information

Supplementary material 1

Supplementary material 2: Flowchart of study participants selection

Supplementary material 3: Histograms showing the population distribution of the AIP

Supplementary material 4: Schoenfeld residual plot of AIP changes over time with All-cause mortality as the dependent variable. The p-value of Schoenfeld Residuals Test result is larger than 0.05 which indicated that AIP is not a time dependent variable and can be analyzed by Cox Proportional Hazards Model

## Data Availability

The datasets used and evaluated in this study can be obtained from the corresponding author upon making a reasonable request.
