# Supplementary material for: Association of atherogenic index of plasma with cardiovascular disease mortality and all-cause mortality in the general US adult population: results from NHANES 2005–2018
Source: Cardiovasc Diabetol. 2024 Jul 16;23:255. doi: 10.1186/s12933-024-02359-z (PMC11253368; doi:10.1186/s12933-024-02359-z)
Supplement: Supplementary file 1 — Supplementary material 1 [file 12933_2024_2359_MOESM1_ESM.docx]

**Table S1. Diagnostic steps for collinearity between AIP and other covariates when All-cause mortality was the dependent variable.**

|  | **VIF** | |
| --- | --- | --- |
|  | Step 1 | Step 2 |
| AIP | 1.2 | 1.2 |
| Age | 1.2 | 1.2 |
| Sex | 1.2 | 1.1 |
| BMI | 1.2 | 1.1 |
| Education | 1.3 | 1.3 |
| Family income-poverty ratio | 1.3 | 1.3 |
| TC | 6.3 | NA |
| TG | 11.2 | NA |
| HDL-C | 7.8 | NA |
| LDL-C | 6.2 | 1.0 |
| Smoking status | 1.1 | 1.1 |
| Drinking status | 1.2 | 1.2 |

VIF: variance inflation factor; VIF = 1/(1-R^2^). Abbreviations as in Table 1.

Note: The variables with VIF>5 will be regarded as collinear variables and can not be included in the multiple regression model.

**Table S2. Exploratory stratified analysis of the associations between AIP index (continuous) and All-cause or CVD mortality.**

|  | **HR (95%CI)** | | | | | |
| --- | --- | --- | --- | --- | --- | --- |
|  | **All-cause mortality** | | | | **CVD mortality** | |
| **AIP index** | **(-1.25, 1.45)** | **<0.0905** | **≥ 0.0905** | ***P*-interaction** | **(-1.25, 1.45)** | ***P*-interaction** |
| **Sex** |  |  |  | 0.861 |  | 0.312 |
| Male | 1.07 (0.87, 1.31) | 0.94 (0.62, 1.41) | 1.31 (0.83, 2.08) |  | 1.27 (0.88, 1.84) |  |
| Female | 1.07 (0.83, 1.38) | 1.03 (0.65, 1.63) | 1.94 (0.97, 3.88) |  | 1.41 (0.89, 2.24) |  |
| **Age, years** |  |  |  | 0.045 |  | <0.001 |
| <40 | 1.10 (0.54, 2.22) | 1.17 (0.54, 2.50) | 0.33 (0.05, 2.12) |  | 2.39 (0.51, 11.27) |  |
| 40-60 | **1.51 (1.08, 2.10)** | 1.09 (0.84, 2.24) | **1.97 (1.06, 3.67)** |  | **2.63 (1.39, 4.98)** |  |
| ≥60 | 0.98 (0.81, 1.18) | 1.01 (0.70, 1.45) | 1.42 (0.86, 2.33) |  | 1.09 (0.78, 1.53) |  |
| **BMI, kg/m^2^** |  |  |  | 0.033 |  | <0.001 |
| <25 | **1.51 (1.12, 1.99)** | 1.13 (0.72, 1.77) | **3.95 (1.65, 9.47)** |  | **2.28 (1.32, 3.93)** |  |
| 25-29 | **1.42 (1.06,1.91)** | **1.98 (1.05, 3.77)** | **2.04 (1.04, 4.12)** |  | 1.47 (0.87, 2.48) |  |
| ≥29 | 1.07 (0.88, 1.30) | 2.98 (0.94, 9.52) | 1.08 (0.62, 1.89) |  | 1.18 (0.84, 1.66) |  |
| **Diabetes** |  |  |  | 0.068 |  | 0.202 |
| Yes | 1.05 (0.82, 1.34) | 1.14 (0.63, 2.06) | **1.68 (1.05, 2.69)** |  | 0.87 (0.57, 1.34) |  |
| No | 0.96 (0.77, 1.18) | 0.90 (0.60, 1.34) | 0.91 (0.48, 1.73) |  | **1.55 (1.04, 2.29)** |  |
| **Hypertension** |  |  |  | 0.576 |  | 0.664 |
| Yes | 1.04 (0.87, 1.25) | 1.15 (0.77, 1.71) | **1.52 (1.00, 2.33)** |  | 1.22 (0.89, 1.67) |  |
| No | 1.13 (0.83, 1.55) | 0.83 (0.46, 1.51) | 1.32 (0.57, 3.07) |  | 1.61 (0.84, 3.06) |  |
| **MetS** |  |  |  | 0.826 |  | 0.329 |
| Yes | 1.05 (0.88, 1.24) | 1.02 (0.71, 1.45) | 1.42 (0.96, 2.09) |  | 1.16 (0.86, 1.56) |  |
| No | 0.91 (0.54, 1.53) | 0.86 (0.47, 1.58) | 4.57 (0.38, 15.25) |  | 1.00 (0.28, 3.44) |  |

Abbreviations: HR: hazard ratios; CI: confidence interval; other abbreviations as in Table 1.

Note: Models adjusted for the same covariates as in model II (Table 3), except for the stratification variable.

**Table S3. Sensitivity analysis after excluding baseline patients with neoplasm and severe heart disease* (N= 14,997).**

|  | No. of case | No. of subjects | HR (95%CI) | *P* value |
| --- | --- | --- | --- | --- |
| **All-cause mortality** |  |  |  |  |
| AIP | 921 | 14,797 | 1.01 (0.78, 1.27) | 0.9912 |
| AIP (quartile) |  |  |  |  |
| Q1 | 170 | 3,751 | Ref. |  |
| Q2 | 244 | 3,746 | 1.06 (0.87, 1.30) | 0.5373 |
| Q3 | 258 | 3,749 | 0.97 (0.79, 1.19) | 0.7830 |
| Q4 | 275 | 3,751 | 1.02 (0.81, 1.30) | 0.9901 |
| **CVD mortality** |  |  |  |  |
| AIP | 260 | 14,797 | 1.15 (0.72, 1.84) | 0.5380 |
| AIP (quartile) |  |  |  |  |
| Q1 | 39 | 3,751 | Ref. |  |
| Q2 | 66 | 3,746 | 1.19 (0.79, 1.77) | 0.3999 |
| Q3 | 89 | 3,749 | 1.37 (0.92, 2.05) | 0.1113 |
| Q4 | 75 | 3,751 | 1.01 (0.72, 1.71) | 0.6082 |

CI: confidence interval.

*: Models adjusted for the same covariates as in model II (Table 3).
