# Supplementary figures and images for: Association of atherogenic index of plasma with cardiovascular disease mortality and all-cause mortality in the general US adult population: results from NHANES 2005–2018
Source: Cardiovasc Diabetol. 2024 Jul 16;23:255. doi: 10.1186/s12933-024-02359-z (PMC11253368; doi:10.1186/s12933-024-02359-z)

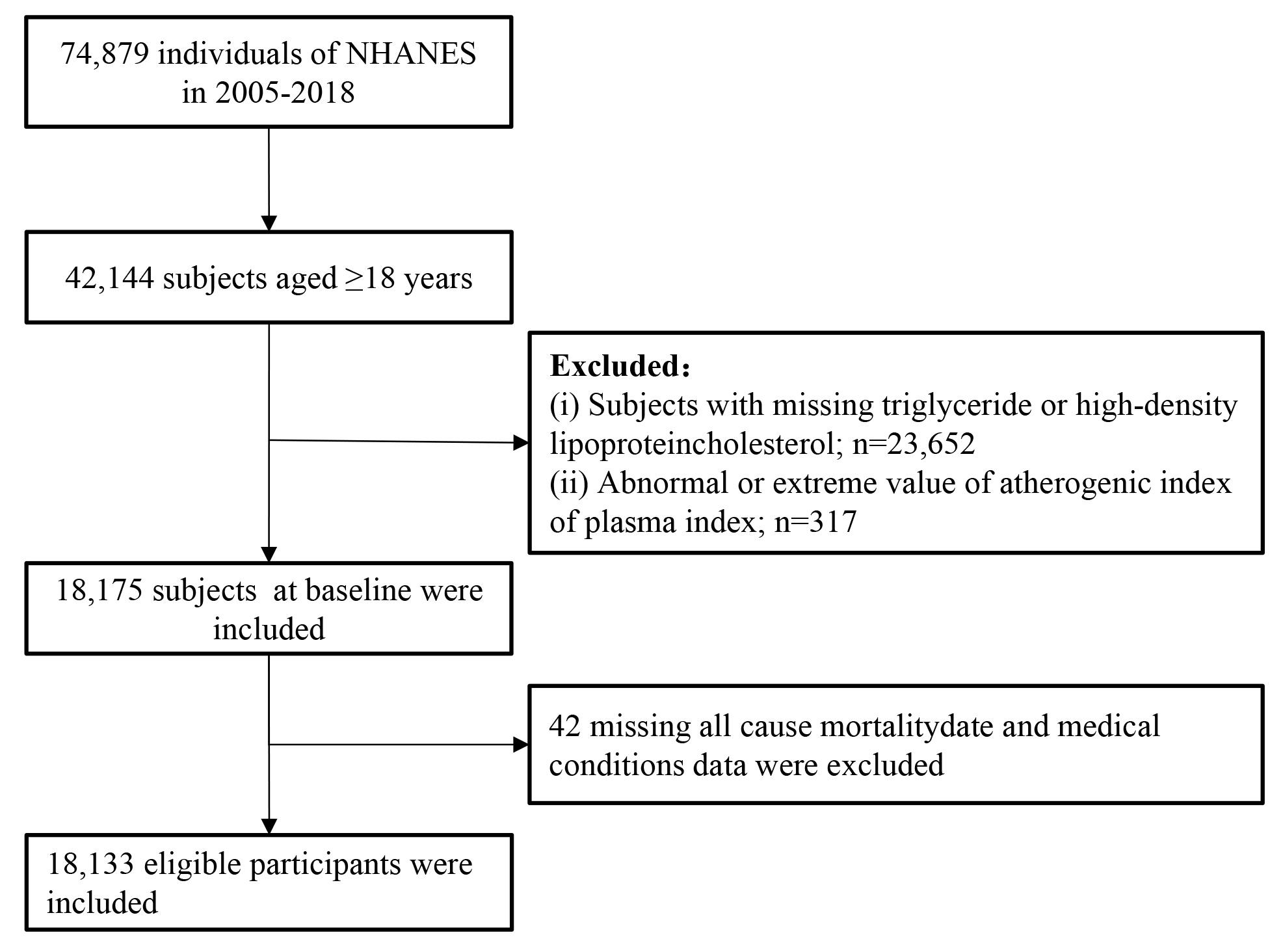

Supplement: Supplementary file 2 — Supplementary material 2: Flowchart of study participants selection [file 12933_2024_2359_MOESM2_ESM.tif]

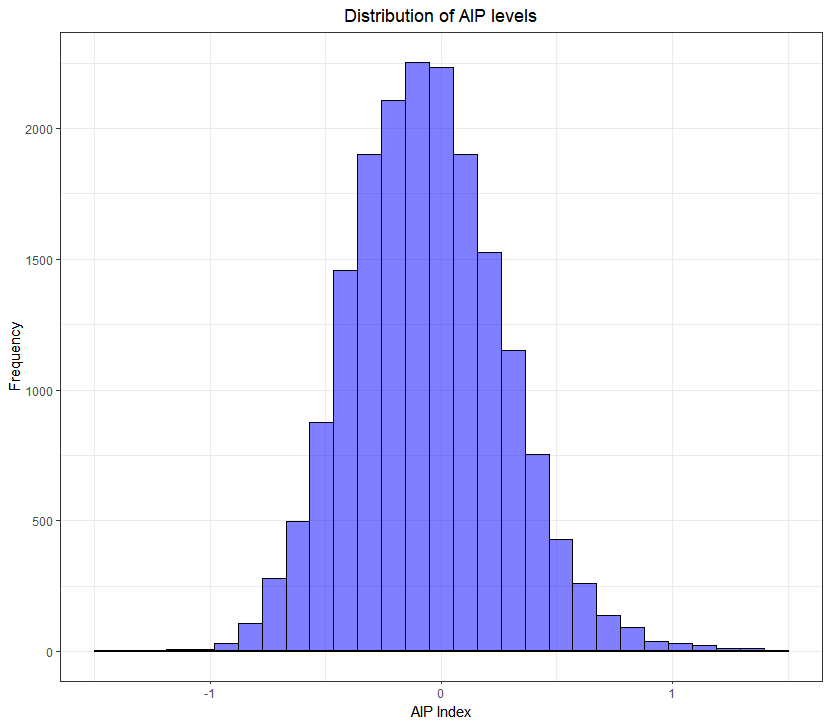

Supplement: Supplementary file 3 — Supplementary material 3: Histograms showing the population distribution of the AIP [file 12933_2024_2359_MOESM3_ESM.tiff]

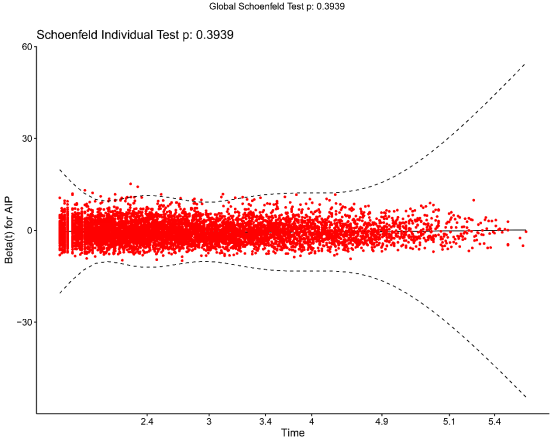

Supplement: Supplementary file 4 — Supplementary material 4: Schoenfeld residual plot of AIP changes over time with All-cause mortality as the dependent variable. The p-value of Schoenfeld Residuals Test result is larger than 0.05 which indicated that AIP is not a time dependent variable and can be analyzed by Cox Proportional Hazards Model [file 12933_2024_2359_MOESM4_ESM.png]
